# Mammary stem cells and progenitors: targeting the roots of breast cancer for prevention

**DOI:** 10.15252/embj.2018100852

**Published:** 2019-06-21

**Authors:** Pirashaanthy Tharmapalan, Mathepan Mahendralingam, Hal K Berman, Rama Khokha

**Affiliations:** ^1^ Princess Margaret Cancer Centre University Health Network University of Toronto Toronto ON Canada

**Keywords:** breast cancer prevention, cell‐of‐origin, high‐risk women, mammary stem and progenitors, progesterone, Cancer, Molecular Biology of Disease

## Abstract

Breast cancer prevention is daunting, yet not an unsurmountable goal. Mammary stem and progenitors have been proposed as the cells‐of‐origin in breast cancer. Here, we present the concept of limiting these breast cancer precursors as a risk reduction approach in high‐risk women. A wealth of information now exists for phenotypic and functional characterization of mammary stem and progenitor cells in mouse and human. Recent work has also revealed the hormonal regulation of stem/progenitor dynamics as well as intrinsic lineage distinctions between mammary epithelial populations. Leveraging these insights, molecular marker‐guided chemoprevention is an achievable reality.

## Introduction

Substantial advances have been made in our understanding of breast cancer etiology, and there is avid interest in cancer prevention. However, molecular‐guided chemoprevention approaches remain limited, and primary preventive options for women at highest risk of breast cancer continue to be centered on irreversible prophylactic surgeries (Hartmann *et al*, 1999; Kauff *et al*, 2002; Eisen *et al*, 2005; Domchek *et al*, 2010; Kotsopoulos *et al*, 2017) that harshly impact quality of life (Guillem *et al*, 2006; NICE, 2013). Chemoprevention, defined by Sporn in 1976, is the use of natural, synthetic, or biological agents to reverse, suppress, or prevent either the initial phases of carcinogenesis as primary prevention or the progression of premalignant cells to invasive disease (Sporn, 1976). Two parameters intrinsic to the success of chemoprevention are that individuals requiring risk reduction measures be identifiable and that treatments to safely, effectively, and precisely target premalignant lesion be well established (Sporn, 1976). Major strides have been made toward achieving the first goal, while the second remains elusive.

Empowered with new understanding in breast biology through the identification, characterization, and regulation of mammary epithelial subpopulations, we are now poised to revolutionize chemoprevention. Normally, mammary stem cells and progenitors give rise to discrete alveolar structures that repeatedly form in the adult breast and their activity is necessary for normal mammary homeostasis. Yet, stem cells and progenitors are considered the cell‐of‐origin in many breast cancers. Therefore, pre‐emptively eliminating these cancer precursors provides the basis for targeted prevention. Here, we review the current state of chemoprevention for high‐risk patients and evidence for mammary stem and progenitors as breast cancer cells‐of‐origin. Beyond estrogen, we outline the mitogenic role of progesterone and its effectors in the adult breast, underscoring their potential in restraining unwarranted mammary cellular expansion. We highlight stem/progenitor molecular vulnerabilities uncovered through OMICs studies and propose a pipeline for discovery of targets for molecular interception. Finally, we discuss some of the challenges and open questions in the complex field of chemoprevention.

## Current chemoprevention in breast cancer

### Defining high‐risk women

Breast cancer continues to be the most frequent cancer in females, affecting about 1 in 8 and causing the greatest number of cancer‐related deaths in women worldwide (Bray *et al*, 2018). Women at high‐risk for breast cancer include those with inherited mutations in the BRCA1 or BRCA2 genes and have a ~ 72 and ~ 69% lifetime risk of developing breast cancer by the age of 80, respectively (Kuchenbaecker *et al*, 2017). A particularly high lifetime risk is also conferred by pathogenic mutations in PTEN (Cowden syndrome, ≥ 25–50%; Tan *et al*, 2012; Evans *et al*, 2018), TP53 (Li–Fraumeni syndrome, 49–90%; Masciari *et al*, 2012; Evans *et al*, 2018), PALB2 (33–58%; Antoniou *et al*, 2014), CDH1 (40–54%; Kaurah *et al*, 2007), and STK11 (Peutz–Jeghers syndrome, 45%; Hearle *et al*, 2006). Other mutations recognized to correlate with increased breast cancer risk occur in genes including ATM, BRIP1, CHEK2, MRE11A, MSH6, NBN, NF1, PMS2, RAD50, RAD51C, and SEC23B; many of which play a role in DNA damage response pathways (Walsh *et al*, 2006; Campeau *et al*, 2008; Antoniou *et al*, 2014; Easton *et al*, 2015; Kurian *et al*, 2017). Large genome‐wide association studies have also exposed genes positively associated with breast cancer susceptibility including FGFR2, TNRC9, MAP3K1, and LSP1, which are related to cell growth and signaling (Easton *et al*, 2007). Finally, genome‐wide single‐nucleotide polymorphism studies have identified > 125 loci associated with genetic breast cancer susceptibility (Easton *et al*, 2007; Milne *et al*, 2017; Evans *et al*, 2018).

**Figure 1:**
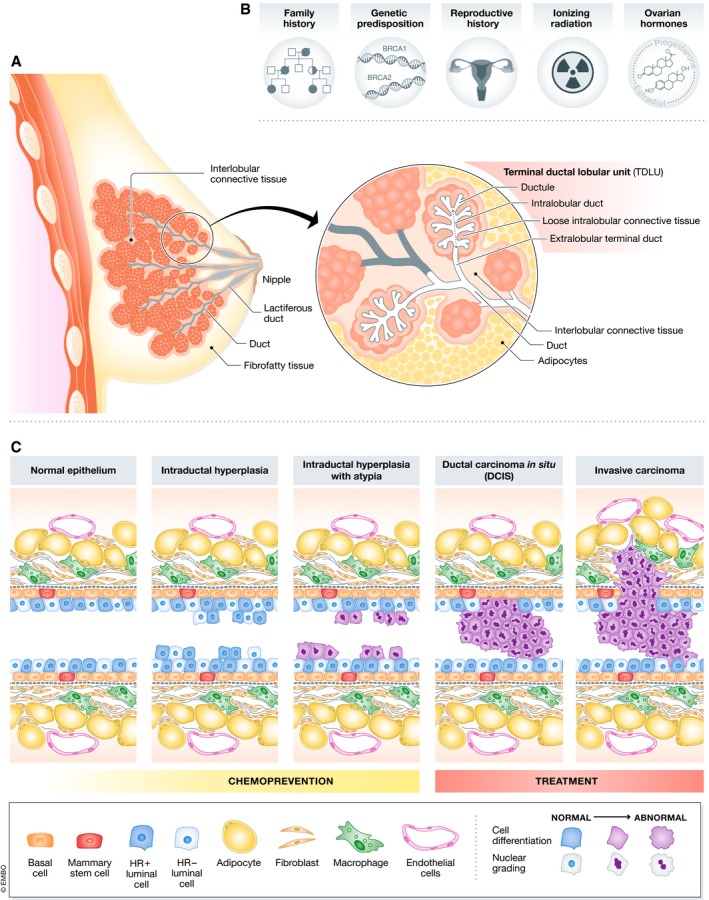
The breast: structure, risk factors and stages of cancer development (A) Schematic of the human breast highlighting terminal ductal lobular units (TDLUs), the site of origin in a number of breast cancers. (B) Some of the major risk factors underlying high‐risk status for breast cancer. (C) Schematic of a ductal cross‐section, depicting the progression of breast cancer from normal bi‐layered epithelium to hyperplasia, to hyperplasia with atypia, to ductal carcinoma *in situ*, and finally to invasive disease.

In addition to genetic predisposition, the following criteria are also used to clinically identify women at high‐risk: (i) first‐degree relative with a breast cancer diagnosis before 50 years of age; (ii) history of atypical hyperplasia; (iii) history of lobular carcinoma *in situ* (LCIS); (iv) chest radiation between 10 and 30 years of age; (v) 5‐year risk of ≥ 1.7% by Gail model; and (vi) lifetime risk of ≥ 20% by International Breast Cancer Intervention Study (IBIS) model (Bevers *et al*, 2018). The Gail model (The Breast Cancer Risk Assessment Tool, BCRAT) is a computer‐based clinical risk assessment tool used to estimate risk in the next 5 years and up to 90 years, while IBIS, BOADICEA, and BRAPRO are examples of other risk prediction models that also incorporate the effects of genetic susceptibility. Recent NCCN guidelines recommend risk reduction strategies for women with a lifetime risk of ≥ 20% (Bevers *et al*, 2018). The ability to pre‐emptively identify women at increased risk for breast cancer is constantly evolving, with an expanding and better‐defined patient pool eligible for chemoprevention, whereas management and risk reduction options are lagging (Fig 1).

### Management strategies for high‐risk patients

Invasive surgery and dated hormonal therapies are the main standard of care options for intervention in women at high‐risk of developing breast cancers. Bilateral prophylactic mastectomy is generally considered for women with very strong family history and/or known gene mutations of high penetrance. It is shown to reduce breast cancer risk by at least 95% in women with BRCA1 or BRCA2 mutation and by 90% for those with a strong family history. Since BRCA1/2 carriers are also at risk of developing highly aggressive ovarian cancers with poor long‐term survival, bilateral salpingo‐oophorectomy (BSO) is also often considered. BSO, which removes the endogenous source of ovarian hormones, has been shown to reduce the risk of breast cancer by 50% in these patient groups (Rebbeck *et al*, 2004; Eisen *et al*, 2005; Domchek *et al*, 2010). In BRCA2 carriers, these effects are mainly observed in the first 3–5 years as they typically present with ER^+^ positive breast cancer and benefit from the immediate hormone deprivation (Lakhani *et al*, 2005; Evans *et al*, 2018). This efficacy of BSO was not seen in BRCA1 carriers who predominantly develop ER‐ cancers, although reduction of contralateral breast cancer with tamoxifen was observed, warranting longer follow‐up in this high‐risk group (Lakhani *et al*, 2005; Evans *et al*, 2018).

Estrogen‐centric strategies continue to dominate the breast cancer prevention field. Currently, the selective estrogen receptor modulators (SERMs) tamoxifen and raloxifene are the first‐line chemopreventive agents (King *et al*, 2001; Fisher *et al*, 2005; Vogel *et al*, 2006, 2010; Goss *et al*, 2011; Cuzick *et al*, 2014, 2015). Tamoxifen is recommended for 5 years to both pre‐ and post‐menopausal high‐risk women, shows a risk reduction of up to 50% and protective effects for up to 20 years after cessation of use. Women at moderate risk are also advised to consider tamoxifen for 5 years, if premenopausal (NICE, 2013). Data compiled from six studies showed tamoxifen is more effective at reducing the incidence of ER^+^ cancers (350 vs. 632 cases) but not the more aggressive ER^−^ breast cancers (173 vs. 144 cases; Fisher *et al*, 2005; Powles *et al*, 2007; Veronesi *et al*, 2007; Goss *et al*, 2011; Cuzick *et al*, 2014, 2015; Narod, 2015). There is also concern as to whether tamoxifen use reduces mortality rates since a reduction in deaths was not observed (Narod, 2015). Moreover, tamoxifen is not recommended for patients with a past history/increased risk of thromboembolic disease or endometrial cancer and has a low compliance rate due to side‐effects (Nelson *et al*, 2013; Padamsee *et al*, 2017). Raloxifene is only recommended to post‐menopausal high‐risk individuals, and a risk reduction of 38% is anticipated. The STAR trial showed that raloxifene, although better tolerated, was inferior for longer term protection compared to tamoxifen (Vogel *et al*, 2010). Other SERMs such as lasofoxifene and arzoxifene have shown similar risk reduction in post‐menopausal women with osteoporosis but these agents have not been introduced into clinical practice (Suh *et al*, 2001; LaCroix *et al*, 2010; Powles *et al*, 2012). Beyond SERMs, aromatase inhibitors (AIs) that block the enzymatic conversion of androgen to estrogen are currently used off‐label for risk reduction but are not FDA approved for chemoprevention (Goss *et al*, 2011; Cuzick *et al*, 2014). High‐risk post‐menopausal women are offered the AI anastrozole or exemestane unless suffering from severe osteoporosis. In the IBISII trial, anastrozole reduced incidence by 50% after 3.5 years of follow‐up while a 60% reduction was observed with exemestane after 2.5 years of follow‐up in the recent MAP3 trial (Goss *et al*, 2011; Cuzick *et al*, 2014). Neither AI is recommended for premenopausal patients.

Other drugs have also been investigated as breast cancer chemopreventive agents in retrospective studies. Bisphosphonates (in osteoporosis trials) were found ineffective in decreasing the risk of invasive post‐menopausal breast cancers after 3–4 years of treatment (Hue *et al*, 2014); fenretinide, a retinoic acid derivative, was tested with second primary breast cancer as the endpoint in two randomized controlled trials with discordant results (Costa *et al*, 2006; Decensi *et al*, 2009). A subsequent randomized placebo‐controlled trial in 2009 (NCT01479192) was terminated early due to slow accrual, possibly due to recognized fenretinide toxicity, highlighting the importance of long‐term tolerability for prevention agents. There have also been serendipitous findings of chemopreventive agents from studying cancer‐related outcomes in patients with co‐morbidities. Diabetic patients taking metformin were found to have an overall decrease in cancer incidence compared to non‐users (Evans *et al*, 2005). Metformin has been reported to decrease the risk of incidence in diabetic women (Bosco *et al*, 2011; Chlebowski *et al*, 2012), whether its preventative effects would be seen in non‐diabetic women is unknown. In addition, multiple clinical studies have demonstrated that statins, the cholesterol decreasing medication, reduce the risk of breast cancer incidence (Cauley *et al*, 2006) and recurrence after diagnosis (Ahern *et al*, 2011). Other recommendations for breast cancer prevention include limiting the duration and dose of hormone replacement therapy, lowering alcohol consumption, maintaining a healthy body weight and lifestyle, and breast‐feeding. Altogether, despite the clinical introduction of breast cancer chemoprevention agents in 1998, tamoxifen remains the only FDA‐approved chemoprevention option available to premenopausal women. Given that high‐risk women develop aggressive tumors before menopause, it is necessary to develop innovative chemoprevention options. A recent review by Evans *et al* (2018) comprehensively delves into the management of high‐risk women.

## New cellular targets in chemoprevention

The hormonal axis has been the preferred target in breast cancer prevention, but evidence pointing to epithelial subsets as the cells‐of‐origin in breast cancer is creating a paradigm shift. Specifically, mammary stem and progenitors are now purported as the cells that undergo transformation (Visvader & Stingl, 2014) and limiting these cancer precursors offers a promising approach (Casey *et al*, 2018). The potential of a cell‐based strategy was recognized early, beginning with pathology studies that highlighted the continuum of breast cancer progression from a premalignant cell to invasive disease (DeOme & Medina, 1969; Wellings *et al*, 1975; Sporn, 1976), which take years to progress. Alterations detected in normal, tumor‐adjacent epithelium that confer oncogenic potential include allelic imbalance, loss of heterozygosity, hypermethylation of promoters (e.g., p16INK4a), upregulation of epigenetic regulators (e.g., EZH2), or aberrant activation of signaling pathways (e.g., p38; Wellings *et al*, 1975; Sporn, 1976; Deng *et al*, 1996; Lakhani *et al*, 1999; Porter *et al*, 2003; Chin *et al*, 2004; Ellsworth *et al*, 2004; Larson *et al*, 2005; Clarke *et al*, 2006; Yao *et al*, 2006; Tripathi *et al*, 2008). Rational development of preventive strategies aimed at cancer precursors necessitates that we understand the cellular composition of the breast as well as the cellular subsets that underlie breast cancer subtypes.

### Defining breast stem cells and progenitor subsets

The adult breast undergoes multiple periods of robust change marked with striking growth and structural remodeling indicative of activated stem/progenitor cell pools. The human breast develops postnatally into an organized ductal tree, and the murine mammary ductal tree is fundamentally similar. In humans, radially branching ducts end in functional pyramidal lobules termed terminal ductal lobuloalveolar units (TDLUs), and analogous lobuloalveolar structures are found in the mouse, making it an instructive model to study the mammary epithelial cell hierarchy and breast cancer. TDLUs ultimately differentiate into milk‐secreting acini during lactation but also repeatedly form and regress over the reproductive lifespan of a female. Specifically, there is a 10‐fold increase in alveoli per lobule and *de novo* lobular formation in pregnancy, as well as significant proliferation during each menstrual cycle, underscoring the gland's regenerative potential (Potten *et al*, 1988; Russo & Russo, 2004). Sustained TDLUs due to incomplete involution either age‐related or post‐gestation are known to increase breast cancer risk. Importantly, TDLUs are thought of as the site(s) of origin for the majority of human breast cancers (Wellings, 1980) and contain stem and progenitor cells (Wellings *et al*, 1975). Microdissected TDLUs from human breast tissue show conserved X inactivation patterns throughout, implying clonal origins, with entire TDLUs originating from the same progenitor (Tsai *et al*, 1996; Diallo *et al*, 2001). Similarly, entire ducts or lobules with identical patterns of loss of heterozygosity have been reported implicating a common progenitor (Lakhani *et al*, 1999). Restricting the appropriate stem/progenitor pool would block cellular expansion, diminish TDLU turnover, and intercept breast cancer establishment at its source.

At the cellular level, both ducts and TDLUs are bi‐layered with two lineages: an inner luminal epithelial layer with cells expressing cytokeratin 8 (K8), K18 as well as hormone receptors (HR, ER/PR), and an outer myoepithelial/basal layer expressing K5, K14, p63, and SMA. Importantly, these two lineages contain defined stem/progenitor‐enriched subpopulations. Cell surface markers used to segregate mammary epithelial cells include MUC1, EpCAM, CD49f, CD24, CD29, CD133, Thy1, CD10, and ALDH in humans and CD24, CD29, CD49f, EpCAM, CD49b, Sca1, Prominin‐1 (human CD133), and CD61 in the mouse (Stingl *et al*, 2001, 2006; Shackleton *et al*, 2006; Eirew *et al*, 2008; Shehata *et al*, 2012). Flow cytometry in conjunction with *in vivo* limiting dilution assays and *in vitro* colony‐forming capacity (CFC) assays has been used to enumerate stem and progenitor activity. Colonies from the human breast have been morphologically scored as basal, luminal, and mixed colonies that likely originate from basal, luminal, and bi‐potent progenitors, respectively. Commonly, EpCAM^−^CD49f^hi^ is used to mark basal cells, EpCAM^+^CD49f^lo^ non‐clonogenic luminal cells, and EpCAM^+^CD49f^hi^ for luminal progenitors, where ALDH^+^ is used specifically to further enrich for progenitors with an alveolar signature and this fraction expresses low levels of luminal cell differentiation (Stingl *et al*, 2001; Eirew *et al*, 2012; Shehata *et al*, 2012). EpCAM^+^MUC1^−^ cells express high K19 and form branched structures similar to TDLUs in 3D cultures and *in vivo*, indicating an enrichment for a TDLU precursor (Gudjonsson *et al*, 2002). Other markers that further segregate luminal cells include GATA3, ErbB3, and ALDH (Asselin‐Labat *et al*, 2007; Ginestier *et al*, 2007; Shehata *et al*, 2012) in humans, while CD14 (Shehata *et al*, 2012), c‐Kit (Lim *et al*, 2009; Regan *et al*, 2012), and Elf5 (Zhou *et al*, 2005; Oakes  *et al*, 2008) segregate the murine luminal compartment. Finally, Lgr5 (Van Keymeulen *et al*, 2011; Plaks *et al*, 2013), Procr (Wang *et al*, 2015), Tspan8 (Fu *et al*, 2017), Dll1 (Chakrabarti *et al*, 2018), and Bcl11b (Cai *et al*, 2017), each further enriches basal subsets for mammary stem cells, although an exclusive mammary stem cell signature remains elusive. Nuances in protocols from tissue dissociation to preferred cell surface markers and stem/progenitor readouts contribute to some discordant findings. Moreover, the potent effect of hormones on the mammary epithelium continues to be overlooked and not controlled for in most studies. However, a recent study has shown that ovarian hormones trigger a heterogeneous cell cycle response in the epithelial subsets (Shehata *et al*, 2018). Similarly, CD61 has been an unreliable luminal progenitor marker but the protein itself is now known to be downregulated by progesterone (Casey *et al*, 2018).

Knowledge of parent–progeny cell relationships and the mammary epithelial hierarchy is important to pinpoint the most effective precursor population to target in prevention. Following orthotopic transplantation *in vivo*, only basal cells possess the functional capacity to generate a full ductal tree, with the ability to both self‐renew and contribute to all subsequent lineages (Shackleton *et al*, 2006; Stingl *et al*, 2006; Spike *et al*, 2012; Wang *et al*, 2015). In the human breast, luminal and basal cells in the same region possess identical chromosomal alterations, implicating a shared ancestry (Deng *et al*, 1996) and single‐cell RNA‐Seq (scRNA‐seq) of primary human breast epithelial cells found a continuous lineage hierarchy that connected the basal lineage to two differentiated luminal branches (Nguyen *et al*, 2018). These studies, together with select lineage tracing reports, document the presence of a bi‐potent basal population (Rios *et al*, 2014; Wang *et al*, 2015). However, other lineage tracing studies challenge this classical view, proposing instead that the combined action of unipotent basal and luminal progenitors maintains the mammary gland (Van Keymeulen *et al*, 2011; Giraddi *et al*, 2015; Wuidart *et al*, 2016; Wang *et al*, 2017). Despite ongoing discussion in this area, a considerable body of work supports specific mammary populations as the candidate cells‐of‐origin for breast cancer subtypes. Finally, techniques such as scRNA‐seq and mass cytometry (CyTOF) are exposing heterogeneity within the basal and luminal compartments (Bach *et al*, 2017; Pal *et al*, 2017; Giraddi *et al*, 2018; Nguyen *et al*, 2018), indicating the necessity of further dissecting precursor–progeny relationships and developmental correlates of tumorigenesis.

### Cells‐of‐origin in breast cancer

Cell‐of‐origin refers to a founder cell that acquires mutations leading to clonal expansion and eventual tumorigenesis (Visvader & Stingl, 2014). Stem and progenitor cells are considered cell‐of‐origin in many cancers, and their defining features of replicative potential and long cellular lifespan render them susceptible to accumulating mutations. It has been recently proposed that variations in cancer risk among tissues can be explained by the number of stem cell divisions (Tomasetti & Vogelstein, 2015; Tomasetti *et al*, 2017). The study's meta‐analysis of relationships between stem cell divisions and the risk of 17 different cancer types across 69 countries supports the concept that lifetime risk strongly correlates with the total number of divisions of the normal self‐renewing cells that maintain tissue's homeostasis (Tomasetti *et al*, 2017). This concept is especially relevant to the breast which undergoes repeated cycles of cell divisions and differentiation throughout the reproductive female lifespan (Fata *et al*, 2000a; Joshi *et al*, 2010). Two‐thirds of mutations in human cancers arise during somatic cell division due to DNA replication errors regardless of environmental factors (Tomasetti *et al*, 2017), and consistent with this, a large proportion of breast cancer risk genes mentioned above are involved in DNA damage repair.

Core pathways in stem cell biology have been identified as key drivers in aggressive breast cancers. Wnt is implicated in self‐renewal (Badders *et al*, 2009; Zeng & Nusse, 2010) and maintains adult stem cells in multiple tissues including the breast. One of the first genes whose ectopic expression was sufficient to induce mammary carcinogenesis, its overexpression in MMTV‐Wnt1 mice (Tsukamoto *et al*, 1988), leads to the expansion of mammary stem cell pools followed by tumorigenesis (Liu *et al*, 2004; Shackleton *et al*, 2006), and its key signaling effector β‐catenin is elevated in > 50% of breast carcinomas (Lin *et al*, 2000). When overexpressed, MMTV‐c‐myc results in amplification of the stem cell compartment where transformation starts in mammary ducts resulting in morphological changes that mimic characteristics of ductal carcinoma *in situ* (Chepko *et al*, 2005). Conversely, signals that decrease the active stem cell pool, such as Tgfb1, correlate with decreased tumorigenesis. Wap‐Tgfb1 mice have fewer mammary stem cells, likely due to premature stem cell aging and senescence, and reduced tumorigenesis even though the number of premalignant lesions remains comparable in hyperplastic outgrowths seen in transplantation assays (Boulanger & Smith, 2001). Finally, beyond absolute stem cell activity, decreased clonal diversity has also been postulated to contribute to age‐related cancer risk. Interestingly, aged mice with compound deletion of protease inhibitors (Timp1 and Timp3) that negatively regulate Notch activation maintain an expanded stem cell pool without increased susceptibility to carcinogen‐induced mammary tumorigenesis (Jackson *et al*, 2015). Hallmarks of aging such as age‐related lobular involution, increased HR^+^ cells, and minor hyperplasia normally detected in aged glands were also absent (Jackson *et al*, 2015). Stem cell loss with aging is postulated to lead to a field of premalignant cells dominated by clones with a proliferative advantage (Klein & Simons, 2011) which has important implications given that age is the primary risk factor in breast cancer, where women experience higher cancer incidence for every decade of life (Kessler, 1992).

Another line of support comes from RNA expression profiling studies where distinct stem/progenitor populations correlate with individual breast cancer subtypes. Of the five commonly accepted subtypes, HR^+^ luminal A and B cancers exhibit a profile similar to that of mature ER^+^PR^+^ luminal cells, although luminal B has a stronger proliferative signature (Cheang *et al*, 2009; Nielsen *et al*, 2010; Prat & Perou, 2011). A recent study that applied a large set of epithelial markers to > 15,000 normal breast cells detected 11 differentiation states for luminal cells and subsequently classified HR^+^ breast cancers into four new subtypes distinct from the current known categories (Santagata *et al*, 2014). Claudin‐low breast cancers have expression profiles similar to the mammary stem cell‐enriched ER‐PR‐ subpopulation (Lim *et al*, 2009; Molyneux *et al*, 2010). Aggressive metastatic triple‐negative (ER^−^PR^−^HER2^−^) breast cancers also show remarkable gene expression similarities to fetal murine mammary stem cells at embryonic days 16 and 18, stages known to have high stem cell capacity (Spike *et al*, 2012; Giraddi *et al*, 2018). Basal‐like breast cancers have expression similarities to ER^−^PR^−^ luminal progenitors (Lim *et al*, 2009; Molyneux *et al*, 2010; Shehata *et al*, 2012). A study has shown that BRCA1 mutation carriers possess an abnormally expanded luminal progenitor pool prior to cancer onset, and mice with tissue‐specific deletion of Brca1 and p53 in the luminal lineage develop mammary cancers that resemble human BRCA1 tumors histologically (Lim *et al*, 2009; Molyneux *et al*, 2010). Luminal progenitors are of immediate interest as the cell‐of‐origin for aggressive cancers in BRCA1 mutation carriers and targeted risk reduction (Al‐Hajj *et al*, 2003; Lim *et al*, 2009; Keller *et al*, 2012; Visvader & Stingl, 2014). It has also been shown that luminal progenitors can give rise to basal‐like breast cancers following oncogenic insults, irrespective of BRCA1 (Koren *et al*, 2015; Van Keymeulen *et al*, 2015; Hein *et al*, 2016). Finally, a population responsible for giving rise to HER2^+^ breast cancers has yet to be pinpointed. This cancer subtype is highly heterogeneous, consisting of both ER/PR^+^ and ER/PR^−^ (Konecny *et al*, 2003; Cancer Genome Atlas Network, 2012). Thus, resolving heterogeneity in each of the subpopulations will help to further tease out additional putative cell(s)‐of‐origin and other non‐stem cells that may serve as cancer precursors (Bu *et al*, 2019; Shehata *et al* 2019).

## Targeting mitogens to limit stem cells and progenitor activity

Just as the knowledge of relevant patient pools for primary prevention is improving, so is our comprehension of breast stem/progenitor populations. Many external cues are now known as crucial regulators of these epithelial subsets during mammary gland homeostasis opening new possibilities to leverage their control over cell‐of‐origin in breast cancer. Progesterone itself is a significant culprit in breast cancer due to its effects on the mammary epithelium (Joshi *et al*, 2015a). However, only ~ 1/3^rd^ of luminal cells are HR^+^, while basal cells, stem cells, and most progenitors lack estrogen/progesterone receptors (ER^−^PR^−^), rendering them dependent on paracrine effectors (Brisken & Duss, 2007; Shehata *et al*, 2012). Chimera transplant studies show that PR null mammary epithelial cells can display alveolar development if supplied with paracrine effectors and we now know that ER^+^PR^+^ cells respond to hormonal cues and in turn stimulate ER^−^PR^−^ cells (Lydon *et al*, 1995; Humphreys *et al*, 1997; Brisken *et al*, 1998).

### Progesterone, thinking beyond estrogen

Physiological, experimental, and population‐based studies all point to progesterone as a powerful mitogen in the adult breast. The Women's Health Initiative (WHI), which followed post‐menopausal women aged 50–79 years in two US multiethnic randomized clinical trials for ~ 20 years, and Million Women's studies report a significantly greater breast cancer risk associated with hormone replacement therapy formulations that also contained progestins compared to estrogens alone (Rossouw *et al*, 2002; Beral, 2003; Chlebowski *et al*, 2015). In the WHI study, the elevated risk persists long‐term post‐intervention (Chlebowski *et al*, 2015). Increased lifetime exposure to ovarian hormones similarly impacts risk, with a higher cumulative number of menstrual cycles correlating to greater risk (Kelsey *et al*, 1993). Circulating progesterone peaks during the luteal phase of the reproductive cycle, a phase when mammographic density has also been observed to be increased by some in the breast (Morrow *et al*, 2010). This is also reflected in mice and can be scored blindly as a function of the estrous cycle. Higher‐order epithelial branching and alveolar mammopoiesis is seen during the progesterone‐high diestrous phase by mammary whole mounts and histology which positively correlates with serum progesterone, not 17β‐estradiol (Fata *et al*, 2000a; Ramakrishnan *et al*, 2002). This highly proliferative progesterone dominant phase, accompanied by differentiation, is followed by marked regression of mammary epithelium, apoptosis, and glandular remodeling. In fact, this transient but repeated physiology generates significant gross changes in the human breast such that histology alone can infer the follicular (progesterone‐low) vs. luteal (progesterone‐high) stage of the menstrual cycle (Fata *et al*, 2000a; Ramakrishnan *et al*, 2002; Hawkins & Matzuk, 2008). A case–control study has shown high‐risk BRCA1/2 mutation carriers have 121% higher serum progesterone levels during the luteal phase compared to non‐carriers (Widschwendter *et al*, 2013). The same study also observed 33% higher estradiol levels and, given that estrogen is required for robust progesterone receptor expression, these increases further underscore the importance of hormone signaling in high‐risk women. Progesterone triggers mammary stem and progenitors activity during each reproductive cycle, when measured by flow cytometry and stem cell assays (Joshi *et al*, 2010, 2015b; Giraddi *et al*, 2015; Shiah *et al*, 2015). A 6‐fold increase in the CD24^lo^CD49f^+^ basal population, a 3‐fold increase in the CD24^hi^CD49f^−^ luminal population, along with a > 10‐fold increase in mammary stem cell activity occur during the progesterone‐high diestrous stage relative to the estrogen‐high estrous stage (Stingl *et al*, 2006; Joshi *et al*, 2010). Similar quantifiable cellular fluctuations have been confirmed in the premenopausal breast, with a significant increase in EpCAM^+^CD49f^hi^ luminal progenitor activity enumerated by CFC assays (Joshi *et al*, 2015b). Given this literature, it is striking that progesterone has been largely overlooked as a target for intervention.

**Figure 2:**
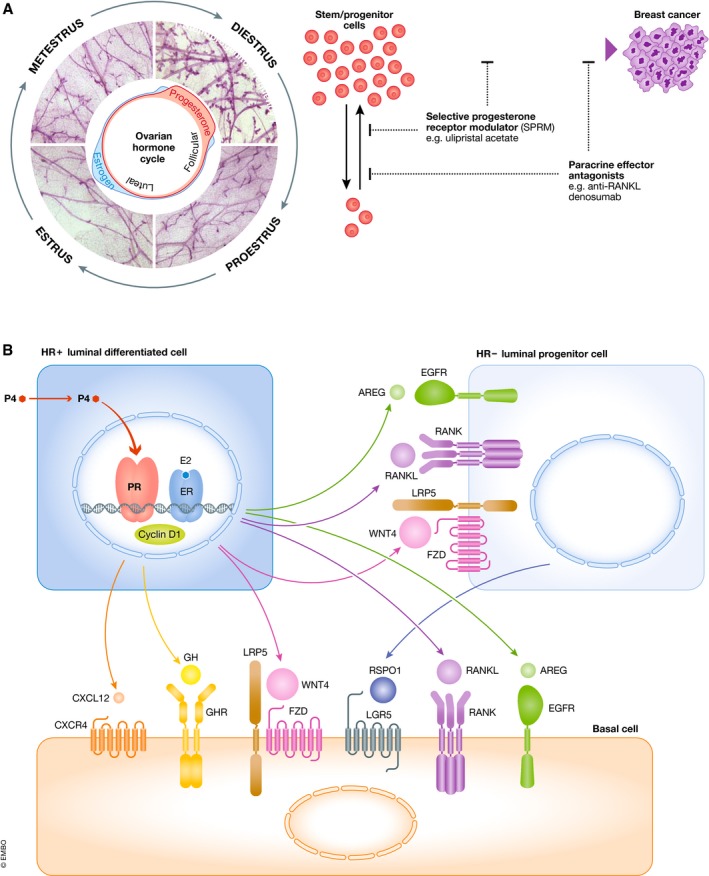
Progesterone‐driven cellular and molecular changes in the mammary gland (A) The murine estrous cycle is shown with mammary whole mounts depicting the gross morphological changes elicited by fluctuations in the ovarian hormones, estrogen, and progesterone. The corresponding expansion in mammary stem and progenitors is also highlighted. Schematic illustrates the strategy of utilizing factors that limit stem/progenitor expansion as chemopreventive agents in breast cancer. Ulipristal acetate is a selective progesterone receptor modulator, and denosumab is an anti‐RANKL agent. (B) Schematic of key mitogenic paracrine effectors downstream of progesterone in the breast, some of which may prove effective as targets in future breast cancer chemoprevention strategies.

Progesterone proves pro‐tumorigenic in both carcinogen‐induced or Brca1 loss‐driven breast cancer pre‐clinical models (Lydon *et al*, 1999; Gonzalez‐Suarez *et al*, 2010; Schramek *et al*, 2010; Tanos *et al*, 2013; Lee *et al*, 2016). Administration of synthetic progestin (medroxyprogesterone acetate, MPA), used to mimic hormone replacement therapy, results in mammary tumors whereas treatment with the PR antagonists mifepristone or telapristone acetate suppresses tumorigenesis (Poole *et al*, 2006; Lanari *et al*, 2009; Lee *et al*, 2016). PR knockout mice similarly show a lower breast cancer incidence (Lydon *et al*, 1999). PR knockout mice also lack normal side‐branching and lobular alveolar development in the adult gland (Lydon *et al*, 1995; Ismail *et al*, 2002). In breast tissue with premalignant atypia, PR isoform (PRA:PRB) ratios are perturbed, with a relative loss of PRB (Mote *et al*, 2002). Tumor‐adjacent normal breast tissue from BRCA1 mutation carriers also has higher PR positivity (Lydon *et al*, 1999; King *et al*, 2004). Clinically, the anti‐progestins mifepristone and onapristone have shown anti‐tumor activity in women with advanced breast cancer although subsequent development was halted due to concerns around liver toxicity. On the other hand, selective progesterone receptor modulators (SPRMs), such as ulipristal acetate, are already in use for emergency contraception, uterine fibroids, or various other gynecological disorders/reproductive problems. These SPRMs are better tolerated, making repurposing for long‐term prevention studies possible which is one of the biggest hurdles in chemoprevention. Ulipristal acetate is currently being tested in a pilot study in premenopausal women at > 17% lifetime breast cancer risk to determine effects on normal breast proliferation and the luminal progenitor population (NCT02408770). Overall, leveraging the potent influence of progesterone and its effects on mammary stem/progenitors, an anti‐progestin approach for prevention offers promise (Fig 2A).

### Progesterone's paracrine effectors

We now know that progesterone acts upon the ER^+^PR^+^ cells, which orchestrates a surge of potent paracrine effectors that in turn stimulate proliferation of the ER^−^PR^−^ progenitor populations (Lydon *et al*, 1995; Humphreys *et al*, 1997; Brisken *et al*, 1998; Joshi *et al*, 2012; summarized in Fig 2B). Though it may have once been thought that these diverse ligands act independently of each other, work to date suggests that they are quite collaborative and converge onto this progesterone‐signaling axis responsible for side‐branching seen during the reproductive cycle and pregnancy. This is also the same axis that has been targeted to inhibit/prevent basal‐like BRCA1 breast cancer. Below we discuss the classical progesterone effectors that can potentially serve as chemoprevention targets such as RANKL, WNT, and amphiregulin (AREG).

RANKL/RANK (receptor activator of NF‐kB ligand/receptor) signaling is a core pathway in the adult mammary gland and breast cancer. It is also a key downstream paracrine effector of progesterone (Gonzalez‐Suarez *et al*, 2010; Schramek *et al*, 2010; Tanos *et al*, 2013; Nolan *et al*, 2016; Sigl *et al*, 2016; Kiechl *et al*, 2017). RANKL‐ and RANK‐deficient mice do not lactate due to defective alveolar development (Fata *et al*, 2000b; Gonzalez‐Suarez *et al*, 2010). Genetic or pharmacological inhibition of RANKL abrogates progesterone‐triggered expansion of mammary stem/progenitors essential for alveologenesis (Fata *et al*, 2000b; Joshi *et al*, 2015b). Blocking RANKL signaling also negates the normal mammary Wnt response which includes the induction of Rspondin in HR^−^ luminal cells (Joshi *et al*, 2015b). Gain‐of‐function studies also show that RANK signaling promotes mammary epithelial cell proliferation and anchorage‐independent growth (Beristain *et al*, 2012). Loss of RANK signaling in breast cancer models drastically limits MPA‐induced and Brca1‐mediated tumorigenesis (Schramek *et al*, 2010; Sigl *et al*, 2016). In otherwise histologically normal tissue of BRCA1 mutation carriers, luminal progenitors positive for RANK expression were shown to be highly proliferative, aberrant in DNA repair, and possessed a basal‐like breast cancer molecular signature (Nolan *et al*, 2016). High circulating RANKL levels correlate with increased breast cancer risk even in post‐menopausal women without a genetic predisposition (Kiechl *et al*, 2017). The RANKL axis is a druggable pathway, effectively inhibited in humans with the monoclonal antibody denosumab. Treatment of breast organoids from pre‐neoplastic BRCA1 heterozygous tissue also resulted in attenuation of progesterone‐induced proliferation (Nolan *et al*, 2016). Building on these pre‐clinical data, a pilot study of denosumab has shown a reduction in proliferation of normal breast cells from women heterozygous for BRCA1 (Nolan *et al*, 2017). It is exciting to note that a breast cancer prevention trial is currently underway to treat BRCA1 mutation carriers with denosumab (BRCA‐P).

STAT5a‐deficient mice phenocopy PR null mice (Miyoshi *et al*, 2001; Cui *et al*, 2004). *In vitro* experiments have shown that progesterone treatment leads to nuclear localization of STAT5a and PR to RANKL enhancer regions. STAT5a null mammary epithelial cells fail to upregulate classical progesterone effectors such as RANKL, WNT4, and AREG in response to the PR agonist R5020 (Obr *et al*, 2013). Work on the inhibition of STAT5 indicates its potential in chemoprevention; reports investigating the progression of early lesions to full‐blown cancers have demonstrated a dependency on STAT5 activation in order to evade apoptosis and pharmacological inhibition of JAK (AG490, ruxolitinib) or STAT5 (C188‐9) led to regression of early lesions (Haricharan *et al*, 2013; Johnston *et al*, 2018). Four weekly injections of C188‐9 also reduced tumor incidence (Haricharan *et al*, 2013), ultimately leading to a prevention clinical trial on the effects of ruxolitinib on premalignant breast cancer (NCT02928978).

The CXCL12‐CXCR4 axis was identified as a ligand–receptor pair in progesterone‐driven transcriptomes of mammary epithelial subsets (Shiah *et al*, 2015). Inhibition of CXCR4 diminished progenitor activity and mammary stem cell frequency (Shiah *et al*, 2015). CXCR4 plays an important role in cancer cell survival in a variety of tissues and is implicated in metastasis. Small molecule inhibitors of CXCR4 are currently being tested in clinical trials with AMD3100 and plerixafor for hematological malignancies (Devine *et al*, 2004; DiPersio *et al*, 2009). Similar to targeting RANK with denosumab, targeting other mitogens involved in stem/progenitor cell maintenance and regulation hold therapeutic promise. Amphiregulin (AREG) is also induced by progesterone, and inhibition of its receptor EGFR (Iressa) abolishes progesterone‐induced terminal end bud formation and proliferation (Fernandez‐Valdivia *et al*, 2008; Aupperlee *et al*, 2013). The metalloprotease ADAM17 is required for AREG shedding and Adam17^−/−^ glands phenocopy those of Egfr null mice (Sternlicht *et al*, 2005). Therefore, in addition to EGFR inhibitors, ADAM17 may be a viable target. Other progesterone targets that may similarly prove therapeutically useful include ID4 (Dong *et al*, 2011) and Cyclin D1 (Said *et al*, 1997).

As mentioned previously, Wnt/B‐catenin signaling is another pathway implicated both in mammary stem/progenitor cell function and mammary tumorigenesis (Li *et al*, 2003; Henry *et al*, 2004; Liu *et al*, 2004). Among Wnt ligands, Wnt4 is a recognized progesterone‐induced factor and Wnt4 knockout glands show decreased side‐branching during early pregnancy (Brisken *et al*, 2000). In transplantation assays, Wnt4^−/−^ epithelium is impaired and reconstitutes only 50% of the fat pad in the first transplant which is lowered to 10% by the third round of transplant, underscoring its requirement in mammary stem cell maintenance (Rajaram *et al*, 2015). It has since been shown that Wnt4 is released from HR^+^ luminal cells and binds its cognate receptor Frizzled on ER^−^PR^−^ progenitors. Targeting Wnt signaling may become a viable option for breast cancer prevention in the future as current phase I and II clinical trials determine the efficacy of Wnt pathway inhibitors such as β‐catenin antagonists (PRI‐724) and anti‐Frizzled agents (vantictumab, OMP‐54F28) in multiple cancer types, as reviewed elsewhere (Takebe *et al*, 2015). Rspondin1 is a Wnt signaling co‐factor shown to mediate mammary stem cell renewal in response to ovarian hormones and in pregnancy. It binds Lgr family proteins that in turn inhibit a negative regulator of Wnt signaling (RNF43/ZNFR3) to sustain Wnt activation. HR‐ luminal progenitors secrete Rspondin1 that acts on basal cells to amplify Wnt signaling during progesterone‐mediated expansion (Cai *et al*, 2014; Joshi *et al*, 2015b). Genetic modulation of Rspondin1 leads to reduced side‐branching, gestational alveologenesis, and mammary stem cell repopulating frequency (Chadi *et al*, 2009; Cai *et al*, 2014). Conversely, administration of recombinant Rspondin rescues Rankl^−/−^ glands. Rspondin3 is expressed in normal basal and stromal cells. Its overexpression in breast cancers corresponds to high levels of a mesenchymal marker, whereas knockdown compromises lactogenic differentiation, xenograft growth, and lung metastasis (Tocci *et al*, 2018). There is currently active interest in developing neutralizing antibodies to various Rspondins (Chartier *et al*, 2016).

## Exploiting mammary lineage vulnerabilities

It is known that tumorigenesis co‐opts normal inherent regulatory networks (Polak *et al*, 2015; Mayers *et al*, 2016; Hoadley *et al*, 2018) and cancers retain characteristics of normal tissues despite acquiring countless mutations (Locke *et al*, 2005). Thus, the molecular identity of mammary cells as dictated by chromatin confirmation, epigenomes, and proteomes will inform the development of rationalized drugs against a mammary lineage or breast cancer subtype. Profiling across multiple genomic platforms (Table 1) is yielding deeper insights into intrinsic differences between the mammary epithelial lineages and has generated powerful reference datasets. These initiatives have uncovered lineage‐specific features of basal and luminal cells, as well as the mammary stem cells and progenitors encompassed within these compartments, for subsequent targeting of cancer precursors.

**Table 1:** Global profiling datasets of mammary epithelial cells

Author	Technique	Populations (time points)	Species	Hormones
Giraddi *et al* (2018)	scRNA‐seq	Fetal (E16, 18), Adult MaSC (10–16 weeks)	Mouse	–
Nguyen *et al* (2018)	scRNA‐seq	Total luminal and basal	Human	–
Pal *et al* (2017)	scRNA‐seq	Total Mammary Gland (2, 5, 10 weeks)	Mouse	EstrusDiestrus
Bach *et al* (2017)	scRNA‐seq	Total EpCAM population	Mouse	Nulliparous (8 weeks)Gestation (14.5 D)Lactation (6 D)Involution (Post 11 D)
Knapp *et al* (2017)	CyTOF	Total epithelium	Human	–
Pal *et al* (2013)	ChIP‐seq (H3K4me3, H3K27me3, H3K9me2)	Adult LP, LM, B (8 weeks)	Mouse	–
Pellacani *et al* (2016)	ChIP‐seq (H3K4me3, H3K4me1, H3K27ac, H3K27me3, H3K9me3, and H3K36me3)WGBS (DNA Methylation)RNA‐seq	LP, LM, B	Human	–
Maruyama *et al* (2011)	ChIP‐seq (H3K4me3, H3K27me3)SAGE‐seq (gene expression)MSDK‐seq (DNA Methylation)	CD24^+^ and CD44^+^	Human	–
Dos Santos *et al* (2015)	WGBS (DNA Methylation)	LP, LM, B	Mouse	Post‐pubertal (nulliparous, 8–15 weeks)Post‐pregnancy (parous, > 12 weeks)
Casey *et al* (2018)	ATAC‐seq (Open chromatin)RRBS (DNA Methylation)UPLC‐MS (Proteomics)	Adult LP, LM, B (8–12 weeks)	Mouse	Hormone pellets
Dravis *et al* (2018)	ATAC‐seq (Open chromatin)RNA‐seqChIP‐seq (H3K27ac)	Fetal MaSC (E18) Adult LM, LP and B (6–10 weeks)	Mouse	–
Gascard *et al* (2015)	RNA‐seqmiRNA‐seqChIP‐seq (H3K36me3)MeDIP‐seq, MRE‐seq, WGBS (DNA methylation)	Myoepithelial, luminal, stem‐like	Human	–
Shiah *et al* (2015)	Microarray	Adult total luminal and basal (8–12 weeks)	Mouse	Hormone pellets

### OMICs‐based lineage distinctions

**Figure 3:**
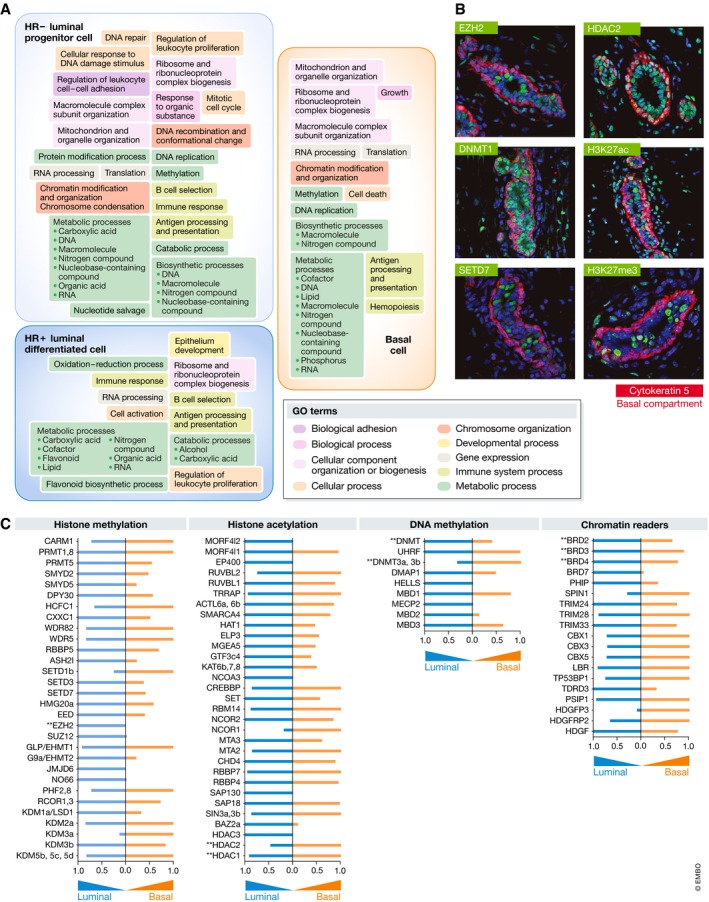
Lineage‐specific molecular programs and epigenetic regulators (A) Schematic illustrating the main GO terms biological processes (≥ 3‐fold upregulated, FDR ≤ 0.01) enriched in the three distinct mammary cell subpopulations in response to progesterone, based on proteomic analysis from Casey *et al*. (B) Visualization of the indicated epigenetic proteins or histone marks (green) *in situ*. Lineage specificity is observed for select proteins (EZH2, DNMT1, and SETD7). Cytokeratin 5 (red) marks the basal compartment. (C) Comparative abundance of epigenetic regulatory proteins detected in luminal vs. basal compartment.

Microarrays of FACS‐purified mouse and human mammary subsets show that the basal and luminal lineages are separate entities (Kendrick *et al*, 2008; Lim *et al*, 2010; Pardo *et al*, 2014; Shiah *et al*, 2015). Kendrick *et al* (2008) found differentially expressed genes in basal (861), HR^+^ (326), and HR^−^ (488) luminal populations, and ovarian hormone‐induced transcriptomes have also been reported (Casey *et al*, 2018). In normal breast epithelium, 255 genes (~ 1.4% of the transcriptome) are differentially expressed between the two phases of menstrual cycle, with ~ 87% of genes being higher in the luteal phase (Pardo *et al*, 2014). Distinct molecular programs are reflected in lineage‐specific expression of key transcriptional factors and signaling components, including the Notch (Raouf *et al*, 2008), WNT (Zeng & Nusse, 2010; van Amerongen *et al*, 2012; Gu *et al*, 2013; Arendt *et al*, 2014), and Hippo pathways (Chen *et al*, 2014; Skibinski *et al*, 2014; Britschgi *et al*, 2017). Proteomic landscapes of murine mammary subpopulations similarly point to lineage‐restricted and progesterone‐driven protein changes (Fig 3; Casey *et al*, 2018). Parallel profiling of open chromatin regions (ATAC‐Seq) and methylomes (RRBS‐Seq) on the same basal and luminal samples permitted quantification of system‐level relationships between chromatin–DNA–RNA–protein states. These data also revealed differential DNA methylation patterns at transcription factor binding sites, identifying motifs hypomethylated and/or enriched in open chromatin regions in basal vs. luminal cells; motifs for key transcription factors included FOXA1, ELF5, GATA3, TP63, known essential regulators of mammary morphogenesis, cell fate, differentiation, and lineage identity. Novel lineage associations were noted for TP53 and EGR1 motifs in basal cells and for FOXA2, SPI1, and FOXP1 motifs in luminal cells (Casey *et al*, 2018). Integration of human breast cell epigenomes and transcriptomes shows that luminal cell genomes harbor more than twice the number of hypomethylated enhancer elements than basal cells (Gascard *et al*, 2015). Unique methylation patterns of breast subsets also illustrate extensive lineage specificity as well as distinct patterns across breast cancer subtypes (Bediaga *et al*, 2010; Holm *et al*, 2010; Gascard *et al*, 2015; Pellacani *et al*, 2016; Casey *et al*, 2018). For instance, in a meta‐analysis of 40 studies, BRCA1 promoter methylation was statistically significantly higher in breast cancers negative for both ER, PR and with a triple‐negative phenotype (Zhang & Long, 2015).

Given the current momentum in utilizing drugs tailored to epigenetic machinery in oncology, understanding the epigenomes of mammary lineages has become an active area (Maruyama *et al*, 2011; Pellacani *et al*, 2016; Dravis *et al*, 2018). Mammary cell proteomes have exposed the enrichment of key epigenetic regulators in the luminal lineage (Casey *et al*, 2018). The study short‐listed 13 rationalized inhibitors corresponding to histone or chromatin modifiers and evaluated their capacity to abrogate luminal and basal CFCs. Specifically, TSA/SAHA (HDAC inhibitors), decitabine (DAC, DNMT1, 3a, 3b inhibitor), and JQ1 (BRD2, 3, 4 & T) stably reduced mammary stem/progenitor function *in vivo*, where DAC also significantly delayed tumor latency and reduced tumor incidence of p53‐driven mammary cancer. Notably, select drugs effectively inhibited the clonogenicity of breast cells from women with BRCA1/2 mutations. Altogether, inhibitors depleted mammary stem/progenitors, delayed aggressive breast cancer tumorigenesis, and demonstrated cytostatic effects underscoring their potential as intervention agents. The histone methyltransferase EZH2 is another candidate for intervention as it is overexpressed in BRCA1 normal breast tissue and implicated in mammary stem cell expansion as well as breast cancer (Ding *et al*, 2006). Trials addressing the clinic utility of epigenetic therapies in breast cancers are already underway. Combinations of HDAC inhibitors with DNMT inhibitors show superior ER re‐expression in breast cancer cell lines, and testing of the HDAC inhibitor entinostat + exemestane is ongoing in HR^+^ breast cancer patients (NCT02115282; Yang *et al*, 2000; Connolly & Stearns, 2012).

### Known intrinsic capacities

In addition to differences in DNA–RNA–protein composition across the two mammary lineages, divergent stress responses and cellular features are observed. For instance, purified human luminal progenitors, which have higher levels of reactive oxygen species (ROS) compared to basal cells, have efficient antioxidant mechanisms and possess higher levels of several glutathione peroxidases (Kannan *et al*, 2014) ROS arise during normal cellular activity but high levels can damage DNA, proteins, and lipids (Gorrini *et al*, 2013), rendering basal cells more vulnerable (Kannan *et al*, 2014). Along the same lines, molecular profiling indicates that luminal progenitors are cells with active transcription. A higher accumulation of R‐loops, which naturally occur as by‐products of transcription, was noted in luminal progenitors compared to basal cells (Zhang *et al*, 2017). This is relevant since luminal progenitors are the likely cell‐of‐origin for BRCA1‐mutated breast cancer, and the dysregulated levels of R‐loops seen in cancers lead to genomic instability (Aguilera & García‐Muse, 2012). Unusually, short telomeres (< 3 kb) were observed in luminal progenitors irrespective of donor age, while contrastingly, basal cells had telomere lengths of ~ 6–8 kb. Only luminal progenitor subsets express human telomerase hTERT, the enzyme responsible for elongating telomere ends, and also express higher levels of several telomere‐associated genes, some crucial for DNA damage repair (MRE11, RAD50, ATM, ATR and BLM; Kannan *et al*, 2013). Oncogene‐induced DNA damage response differs across human luminal and basal populations; luminal subset exhibits copious DNA damage and repair activation (more γH2AX & 53BP1 foci), whereas basal cells show little response (Morel *et al*, 2017). Thus, luminal progenitors appear to be better equipped with DNA damage machinery, ultimately impacting their ability for transformation. Other findings suggest that select basal cells are the most likely cell‐of‐origin for a subclass of triple‐negative breast cancers with low chromosomal instability and mutational load (Morel *et al*, 2017). PARP inhibitors specifically leverage DNA damage responses to induce synthetic lethality; therefore, lineage‐specific repair capacities hold clinical value. Overall, recognizing lineage‐imposed differences will guide the generation of appropriate intervention strategies against specific cells‐of‐origin.

## Molecular‐guided prevention pipeline

**Figure 4:**
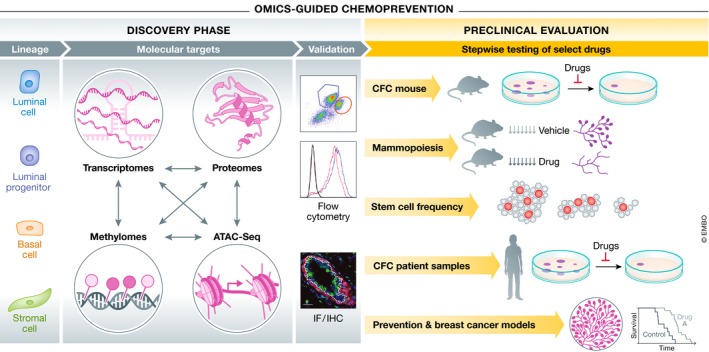
A path to OMICS‐guided chemoprevention A workflow modeling a discovery‐to‐intervention pipeline for OMICs‐guided chemoprevention. FACS‐purified mammary cell populations are the input for integrative molecular profiling, target validation, rationalized drug identification, and evaluation in a series of biological, pre‐clinical assays.

Table 1 summarizes the current data resources available to the breast research community that span breast mammopoiesis. From fetal mammary stem cells to the adult gland under defined hormone settings, these bodies of work provide a foundation for the discovery of novel chemopreventive agents using a systems approach to mammary cell biology (Fig 4). Mining these datasets (genomes, epigenomes, and proteomes) aids to unravel the molecular nature of distinct cells‐of‐origin within the breast. Specifically, these data help expose novel and/or unique biological pathways and highlight putative vulnerabilities intrinsic to specific mammary subpopulations. The corresponding pharmacological agents that are subsequently identified require successive evaluation in rigorous experiments for functional and therapeutic validation pertaining to distinct aspects of breast cancer development.

Utilizing stem cell function (clonogenicity, *in vivo* mammopoiesis, and limiting dilution) and tumor onset, vital pre‐clinical data can be generated. For instance, mouse or human breast epithelial cell 2D colony‐forming capacity assays are a simple, cost‐effective method to screen for drugs that decrease clonogenicity. Excitingly, 3D organoids have also been reported that form bi‐layered morphological structures mimicking the complexity of breast terminal ductal lobular units. Although these culture systems are still being optimized, these structures have been reported as exclusively arising from bi‐potent stem/progenitor cells in the basal compartment and may prove useful as screening tools similar to 2D colony assays (Linnemann *et al*, 2015; Sokol *et al*, 2016). Casey *et al* showed how *in vivo* mammopoiesis assays that measure effects on progesterone‐driven side‐branching and lobuloalveolar development (the putative sites of tumorigenesis) can also be successfully used to further short‐list agents with potential in chemoprevention and ultimately limit cancer incidence in breast cancer models. Limiting dilution assays that enumerate mammary repopulating potential can similarly pinpoint agents effective against mammary stem cells, the putative cell‐of‐origin in select breast cancers. Finally, a number of informative breast cancer models exist, yet are unsuitable for the study of chemoprevention due to their overtly aggressive nature. Genetically engineered mouse models deficient in genes such as Brca1 and p53 exhibit pre‐neoplastic events such as increased numbers of mammary stem/progenitors and hyperplasia followed by multiple mammary tumors (Brodie *et al*, 2001; Evers & Jonkers, 2006). Evaluating drug efficacy in limiting these pre‐neoplastic events provides essential evidence necessary to accelerate translation into clinical trials, as previously demonstrated (Nolan *et al*, 2016; Sigl *et al*, 2016). This overarching workflow is depicted in Fig 4.

## Open questions

In the upcoming years, new approaches to breast cancer prevention are bound to flourish. The World Health Organization Global Action Plan for the Prevention and Control of Noncommunicable Diseases hopes for a 25% reduction in cancer mortality rates by 2025, and the United Nations Sustainable Development Goals program strives for a 33% reduction by 2030, although < 10% of current research funding is dedicated to prevention research (Song *et al*, 2018). Outlining the contributions of lineage and hormones, this review highlights how relevant mammary biology empowers identification of new therapeutic targets to limit unwarranted stem cells and progenitors, the seeds of transformation. However, barriers still hinder wide clinical implementation of chemoprevention, from the patient, clinical, and research standpoint.

Ensuring minimal disruption to reproductive health and drug cytotoxicity is essential in breast cancer chemoprevention. How long should a patient receive a drug and how often? High‐risk groups have an increased lifetime risk so when should treatment begin? Challenges of balancing long‐term compliance against lifetime risk call for the development of new intermittent drug regimens. Stalling expansion of stem and progenitor cell populations in a reversible manner may allow normal activity such as lactation. Anti‐progestins may address these needs as their use can be restricted to a specific reproductive phase. Further, repurposing drugs with known safety profiles, such as ulipristal acetate, denosumab, and metformin, allow immediate translation.

From a clinical standpoint, important questions center on how to design prevention trials. What would be considered a benchmark of effective prevention? Some interesting questions remain if it is sufficient to stabilize breast cancer precursor populations vs. eradicating them? Careful consideration of how to define appropriate clinical endpoints of therapeutic response in prevention will require further investigation. Are there surrogate indicators that can be used to monitor prevention? There is an unmet need for better surrogate indicators, especially for trials of agents with poorly established potential for delayed/long‐term toxicities. Innovation in non‐invasive molecular imaging and liquid biopsy‐based monitoring may resolve some of these challenges.

At the basic level, heterogeneity of adult breast tissue as a function of age, reproductive history, and ethnicity continues to pose challenges. How do other known risk factors, such as obesity, relate to breast cancer cells‐of‐origin? Given the sheer amount of non‐epithelial components in the breast, such as stroma, adipocytes, and immune cells, what is their influence on the nature of risk and their role as targets of prevention? Interestingly, a recent study demonstrates a mesenchymal adipocyte contribution to the mammary epithelium (Joshi *et al*, 2019). Moreover, as we continue to dissect cellular heterogeneity within normal epithelium and breast cancers, the cell‐of‐origin field will correspondingly move forward and further advance molecular‐guided chemoprevention.

## Conflict of interest

The authors declare that they have no conflict of interest.
